# Relationship between teaching modality and COVID-19, well-being, and teaching satisfaction (campus & corona): A cohort study among students in higher education

**DOI:** 10.1016/j.puhip.2021.100187

**Published:** 2021-08-27

**Authors:** Atle Fretheim, Arnfinn Helleve, Borghild Løyland, Ida Hellum Sandbekken, Martin Flatø, Kjetil Telle, Sara Viksmoen Watle, Alexander Schjøll, Sølvi Helseth, Gro Jamtvedt, Rannveig Kaldager Hart

**Affiliations:** a Faculty of Health Sciences, Oslo Metropolitan University, PO Box 4 St. Olavs Plass, N-0130, Oslo, Norway; b Centre for Pandemic Interventions Research, Norwegian Institute of Public Health, PO Box 222 Skøyen, N-0213, Oslo, Norway; c Centre for Evaluation of Public Health Measures, Norwegian Institute of Public Health, PO Box 222 Skøyen, N-0213, Oslo, Norway; d Centre for Fertility and Health, Norwegian Institute of Public Health, PO Box 222 Skøyen, N-0213, Oslo, Norway; e Health Services Division, Norwegian Institute of Public Health, PO Box 222 Skøyen, N-0213, Oslo, Norway; f Infection Control and Environmental Health, Norwegian Institute of Public Health, PO Box 222 Skøyen, N-0213, Oslo, Norway; g Centre for Welfare and Labour Research, Oslo Metropolitan University, PO Box 4 St. Olavs Plass, N-0130, Oslo, Norway

**Keywords:** COVID-19, Infection control, Public health, Student health, Well-being, In-person

## Abstract

**Objectives:**

Higher education institutions all over the world struggled to balance the need for infection control and educational requirements, as they prepared to reopen after the first wave of the COVID-19 pandemic. A particularly difficult choice was whether to offer for in-person or online teaching. Norwegian universities and university colleges opted for a hybrid model when they reopened for the autumn semester, with some students being offered more in-person teaching than others. We seized this opportunity to study the association between different teaching modalities and COVID-19 risk, quality of life (subjective well-being), and teaching satisfaction.

**Study design:**

Prospective, observational cohort study.

**Methods:**

We recruited students in higher education institutions in Norway who we surveyed biweekly from September to December in 2020.

**Results:**

26 754 students from 14 higher education institutions provided data to our analyses. We found that two weeks of in-person teaching was negatively associated with COVID-19 risk compared to online teaching, but the difference was very uncertain (−22% relative difference; 95% CI -77%–33%). Quality of life was positively associated with in-person teaching (3%; 95% CI 2%–4%), as was teaching satisfaction (10%; 95% CI 8%–11%).

**Conclusion:**

The association between COVID-19 infection and teaching modality was highly uncertain. Shifting from in-person to online teaching seems to have a negative impact on the well-being of students in higher education.

## 1 Introduction

Higher education institutions around the world shut down during the first half of 2020, when the first wave of the COVID-19 pandemic struck. A difficult question, as institutions prepared to reopen, was whether they should switch from in-person to online teaching [1].

As Gressman and Peck put it: “In the absence of relevant prior experience, these institutions are largely in the dark about how one might expect a COVID-19 outbreak to evolve in the unique environment of a college campus and how much of an effect the many possible mitigation strategies should be expected to produce.” [2].

Early on, attempts were made at modelling the risk of offering in-person teaching on campus [2,3], e.g. a group at Cornell University concluded that shifting to online teaching would lead to more COVID-19 cases, than a full return of students. This finding was premised on intense surveillance with regular testing of everyone on campus every 5 days [3].

Brauner and colleagues made use of the variation in timing of non-pharmaceutical interventions across 41 countries from January to May 2020, to disentangle the impact of individual interventions. They found that concurrent closing of schools and universities was associated with a 38% (95% CI 16%–54%) reduction of the effective reproductive number [4]. As this was a retrospective, observational study, the findings may be confounded by unobserved factors.

When universities and colleges across the world started to reopen after the first pandemic wave, typically in August/September 2020, they opted for different teaching models [5]. This variation has been utilised in a handful of studies from the United States that seem to show that transmission has been higher in counties hosting institutions offering in-person teaching than in counties where institutions mainly offered online teaching [[6], [7], [8], [9]]. Whereas these studies have shown associations using county-level data, this is the first study to our knowledge of the individual impacts of shifting from in-person to online teaching in higher education.

Apart from these American studies, remarkably little empirical work has been done to assess the consequences of shifting from in-person to online teaching in higher education as an infection control measure.

While the evidence base is weak for shifting from in-person to online teaching to control the spread of COVID-19, it is even weaker for other outcomes, such as well-being and quality of life.

In Norway, higher education institutions opted for a hybrid model when they reopened for the autumn semester in August 2020, by offering more online teaching to some students and more in-person teaching to others, e.g. first year students. The variation across students and over time in teaching modality gave us an opportunity to study the relationship between exposure to in-person versus online teaching, and key outcomes.

Our aim with this study was to assess the association between teaching modality (in-person or online teaching), and COVID-19, well-being, and satisfaction with teaching, among higher education students in Norway.

## 2 Methods

Our reporting adheres to the STROBE-statement. We registered the study in advance at ClinicalTrials.gov (Identifier: NCT04529421) and published the study protocol with analysis plan before recruitment started [10].

The study took place from September to December 2020. All individuals who were registered as students at the participating institutions were invited by SMS and/or e-mail. The invitation included a link to a web-based informed consent form and a questionnaire.. The invitation included a link to a web-based informed consent form and a questionnaire. We sent two reminders. Students who consented, received a link to the questionnaire every two weeks during the study period, i.e. up to 8 times.

We had developed the questionnaire through an iterative process, partly based on items from existing questionnaires. Pilot testing was done with a group of 10 students at Oslo Metropolitan University.

The questionnaire contained items on how much in-person and online teaching they had been offered over the last two weeks, and on testing for COVID-19, subjective well-being, satisfaction with teaching, social activities, and more (see Supplement 1).

The participants consented to linking their survey data to the administrative data system for higher education in Norway, which gave us access to information about which study program each student was enrolled in, basis for admission, study status, academic results, gender, and age (see Supplement 2).

Before data analysis, we decided to limit our analyses to students registered as 1st, 2nd, or 3rd year bachelor level students. The reason was that we realised, based on feed-back from participants, that the study was of limited relevance for many master level students as they have fewer scheduled teaching sessions and spend more time writing theses, than undergraduate students..

### 2.1 Primary outcome

• COVID-19 incidence (self-reported positive test results)

### 2.2 Secondary outcomes

• Well-being (“Overall, how satisfied are you with life right now?” on a 0–10 scale)
• Teaching satisfaction (“Overall, how satisfied have you been with the teaching you have received in the past 14 days?” on a 0–10 scale)
• COVID-19 testing (self-reported)
• Quarantine (self-reported)

We added the question about quarantine after we published the protocol. Data on learning outcomes are not yet available and will be reported at a later stage.

We defined in-person teaching as a continuous variable:

$$In-person\;teaching=\frac{Number\;of\;days\;offered\;in-person\;teaching}{Number\;of\;days\;offered\;in-person\;teaching+Number\;of\;days\;offered\;online\;teaching}$$

### 2.3 Analytical approach

We ran multivariate regressions (ordinary least squares) adjusting for a range of potentially confounding variables, both time constant (institution, year and field of study (and their interaction) parents’ country of origin, own country of origin, gender, age, age squared, parents’ educational level) and time varying (number of roommates, home ownership, total proportion in quarantine at institution, alcohol consumption, use of public transport, total amount of offered teaching, and number of hours of paid work, and survey round).

We realised, during the analysis phase that we needed to account for coinciding infection control measures. To this end, we included the proportion of study participants (excluding the individual) at the same institution who were in quarantine, as a proxy control variable.

We collected exposure and outcome data from the same response questionnaire. It is therefore possible that the outcome (positive COVID-19 test) happened before the exposure (teaching modality) in some cases.

Finally, we also ran analyses using self-reported actual on campus presence as the independent variable.

### 2.4 Sensitivity analyses

We conducted the following sensitivity analyses to check the robustness of our main findings:

- Comparing the quartile with most in-person teaching to the quartile with least
- Changing the exposure variable from continuous (proportion of in-person teaching) to dichotomous (>80% in-person teaching, or not)
- Restricting data for COVID-19 related outcomes to data from the period immediately following the period when data on teaching modality was collected (lag model)
- Our basic model included a set of dummy variables (fixed effects) for institution and survey round. These variables net out, respectively, institution specific characteristics that are constant over time, and time specific factors shared across institutions. As a sensitivity test, we included a full set of interactions between the survey round and institution dummies, replacing proportion of students in quarantine. This interaction will capture any change that affects one institution at one time point, regardless of the nature of this change.
- Using logit and negative binomial models to check the robustness of our findings for positive COVID-19 tests, testing, and quarantining.

As a robustness check we also ran a lead model, i.e. we tested for associations between data on outcomes preceding data on teaching modality. A statistically significant (p < 0.05) association between outcomes and future predictors would indicate that confounders are present.

Recognizing the potential importance of coinciding infection control measures as confounding variables, we decided to explore whether controlling for county level COVID-19 incidence in the week preceding each survey round would influence our findings.

We also decided to conduct a supplementary fixed effects analysis, i.e. assessing the association between teaching modality and outcomes based on the variation we observed for each individual participant, instead of comparing across participants.

### 2.5 Power analysis and sample size

We based our power calculation on the assumption that 0.23% (230 per 100.000) of the participants with predominantly online teaching would report testing positive for COVID-19, over a 10-week intervention period – corresponding to the incidence for the age group 20–29 in Norway at the time. To detect an effect of in-person teaching corresponding to a doubling of COVID-19 risk, we estimated a need for 21 000 respondents to be 80% certain to detect an effect at the 5% significance level.

## 3 Results

The leadership at 14 universities and university colleges, representing around 45% of all students in higher education in Norway, agreed to take part in the study [11].

**Fig. 1:**
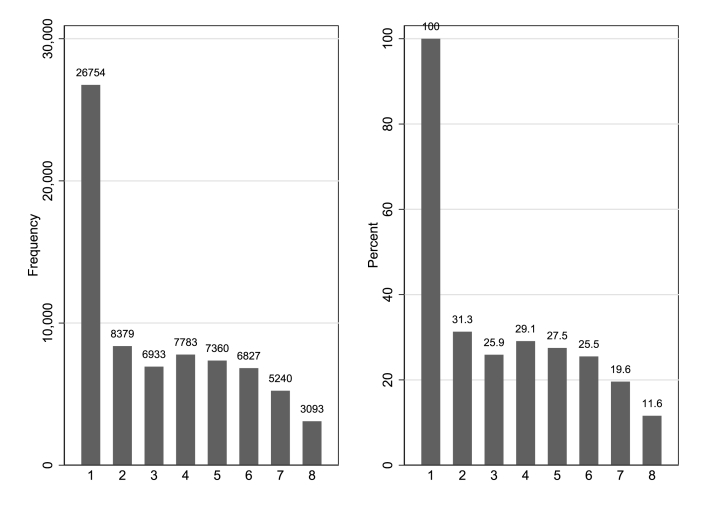
Attrition and number of respondents over survey rounds.

We invited all 142 384 registered students at the 14 institutions. 35 423 of these students responded to the survey at least once. We excluded part-time students and students that were not 1st, 2nd, or 3rd year students. This left us with 26 754 participants that we included in our analyses. The number of responses per survey round is shown in Fig. 1. The total number of responses was 72 369, meaning that we collected around 5 ½ weeks’ worth of data per participant, on average.

**Fig. 2:**
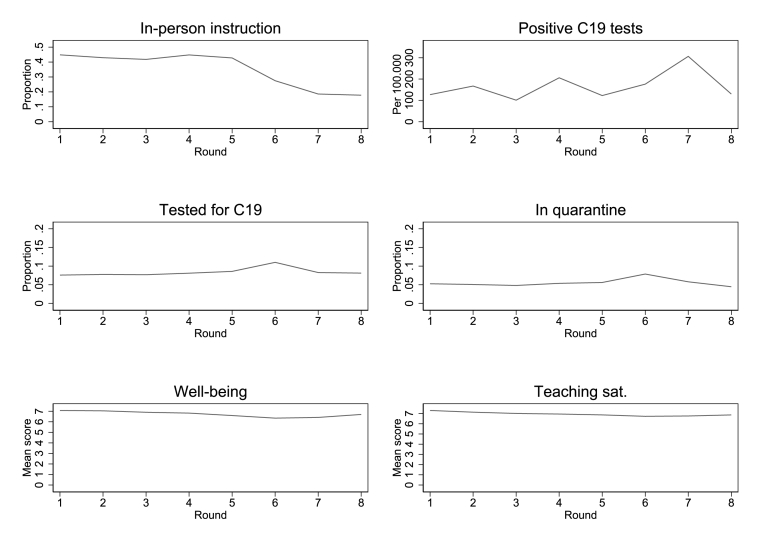
Trends in main outcomes over survey rounds.

Fig. 2 is a presentation of how teaching modality and outcomes changed during the study period.

Key background variables are shown in Table 1, where we have divided the participants into quartiles according to the proportion of in-person teaching they were offered. Results from balancing tests are shown in eTable 1. The difference across modality quartiles was statistically significant (p < 0.05) for gender, parental education, and migration background, but became insignificant after controls for year and field of study were added. For age, this difference persisted also when all the association was purged of other observed covariates.

**Table 1:** Descriptive statistics for background variables.

Variable			By quartile of proportion in-person teaching			
	Category	All	Q1	Q2	Q3	Q4
Gender	Female	18 244 (68.2%)	3404 (70.7%)	4939 (71.5%)	4896 (66.6%)	5005 (65.1%)
	Male	8510 (31.8%)	1412 (29.3%)	1968 (28.5%)	2451 (33.4%)	2679 (34.9%)
Parents higher education	No	8070 (30.2%)	1444 (30.0%)	2115 (30.6%)	2234 (30.4%)	2277 (29.6%)
	Yes	18684 (69.8%)	3372 (70.0%)	4792 (69.4%)	5113 (69.6%)	5407 (70.4%)
Respondents’ place of birth	Norway	22811 (85.3%)	3930 (81.6%)	5956 (86.2%)	6285 (85.5%)	6640 (86.4%)
	Europe	1774 (6.6%)	374 (7.8%)	398 (5.8%)	489 (6.7%)	513 (6.7%)
	Africa	422 (1.6%)	109 (2.3%)	113 (1.6%)	107 (1.5%)	93 (1.2%)
	Asia	1338 (5.0%)	290 (6.0%)	357 (5.2%)	359 (4.9%)	332 (4.3%)
	Australia/Oceania	17 (0.1%)	6 (0.1%)	5 (0.1%)	2 (<1%)	4 (0.1%)
	North America	185 (0.7%)	53 (1.1%)	31 (0.4%)	52 (0.7%)	49 (0.6%)
	South or Central America	165 (0.6%)	47 (1.0%)	41 (0.6%)	35 (0.5%)	42 (0.5%)
	Not reported	42 (0.2%)	7 (0.1%)	6 (0.1%)	18 (0.2%)	11 (0.1%)
Parents’ place of birth	Norway	20177 (75.4%)	3504 (72.8%)	5210 (75.4%)	5540 (75.4%)	5923 (77.1%)
	Europe	3036 (11.3%)	580 (12.0%)	721 (10.4%)	850 (11.6%)	885 (11.5%)
	Africa	670 (2.5%)	159 (3.3%)	191 (2.8%)	181 (2.5%)	139 (1.8%)
	Asia	1922 (7.2%)	307 (6.4%)	571 (8.3%)	545 (7.4%)	499 (6.5%)
	Australia/Oceania	35 (0.1%)	11 (0.2%)	10 (0.1%)	5 (0.1%)	9 (0.1%)
	North America	325 (1.2%)	74 (1.5%)	66 (1.0%)	87 (1.2%)	98 (1.3%)
	South or Central America	253 (0.9%)	61 (1.3%)	74 (1.1%)	62 (0.8%)	56 (0.7%)
	Not reported	336 (1.3%)	7 (0.1%)	6 (0.1%)	18 (0.2%)	11 (0.1%)
Age (SD)		25.14 (6.84)	26.56 (7.63)	24.61 (6.36)	24.94 (6.83)	24.91 (6.64)
Year of study (SD)		1.61 (0.76)	1.76 (0.80)	1.63 (0.75)	1.55 (0.73)	1.56 (0.74)
Previous positive COVID-19 test (SD)		127.1 (3562.7)	207.6 (4552.5)	144.8 (3802.5)	81.7 (2586.8)	104.1 (3225.2)
Hours of paid work last 14 days (SD)		14.6 (18.4)	17.5 (20.9)	14.8 (17.7)	14.0 (18.0)	13.2 (17.6)
N		26 754	4816	6907	7347	7684

Table 2 shows outcomes measured throughout the observation period. A total of 112 participants reported having had a positive COVID-19 tests. We found the highest incidence of COVID-19 in the quartile with least in-person teaching offered (206 cases per 100 000), and the lowest incidence in the quartile with most in-person teaching (123 per 100 000). Quarantining and testing followed the same pattern, with 9% and 6% being tested and quarantined respectively, in the quartile offered least in-person teaching. For the quartile offered most in-person teaching, the corresponding figures were 7% and 5%, respectively. Well-being and teaching satisfaction were lowest in the quartile with least in-person teaching (6.5 on a scale from 0 to 10 for both outcomes), and highest in the top quartile (7.2 and 7.8 for teaching satisfaction and well-being, respectively).

**Table 2:** Descriptive statistics for outcome variables. Means and standard deviations.

Variable	All	By quartile of proportion in-person teaching			
		Q1	Q2	Q3	Q4
Positive test (per 100 000)	155.0 (3934.4)	206.3 (4536.9)	149.8 (3867.8)	134.6 (3666.8)	123.3 (3505.6)
Tested	0.08 (0.27)	0.09 (0.29)	0.09 (0.29)	0.08 (0.26)	0.07 (0.26)
Quarantined	0.05 (0.23)	0.06 (0.24)	0.06 (0.24)	0.05 (0.22)	0.05 (0.21)
Satisfied with teaching	7.06 (2.52)	6.53 (2.58)	6.55 (2.44)	7.35 (2.30)	7.83 (2.49)
Well-being	6.85 (2.33)	6.54 (2.40)	6.66 (2.32)	7.00 (2.27)	7.21 (2.27)
N	72 243	19 879	16 687	17 827	17 850

### 3.1 Main findings

Our main findings are presented in Table 3.

**Table 3:** Main results, coefficients (95% confidence intervals) from ordinary least squares analyses. Relative scale is obtained by dividing the coefficient and their standard error on the average of the outcome when the explanatory variable takes zero.

	Absolute scale		Relative scale	
	Unadjusted	Adjusted	Unadjusted	Adjusted
	**COVID-19 positive**			
Proportion in-person	−91.0*	−49.6	−41.2*	−22.4
	(-171.6 to −10.4)	(-171.0 to 71.8)	(-77.7 to −4.7)	(-77.4 to 32.5)
N	72 243	69 930	72 243	69 930
	**COVID-19 tested**			
Proportion in-person	0.0***	0.0	−25.7***	−3.7
	(0.0–0.0)	(0.0–0.0)	(-32.4 to −19.0)	(-13.2 to 5.8)
N	72 369	70 055	72 369	70 055
	**Quarantine**			
Proportion in-person	0.0***	0.0	−28.1***	−11.0
	(0.0–0.0)	(0.0–0.0)	(-36.5 to −19.6)	(-23.0 to 1.1)
N	72 369	70 055	72 369	70 055
	**Satisfied with teaching**			
Proportion in-person	1.6***	1.0***	24.4***	15.7***
	(1.5–1.7)	(0.9–1.1)	(23.2–25.6)	(14.3–17.1)
N	71 679	69 391	71 679	69 391
	**Well-being**			
Proportion in-person	0.8***	0.4***	12.1***	6.8***
	(0.7–0.9)	(0.4–0.5)	(11.0–13.2)	(5.5–8.1)
N	72 110	69 839	72 110	69 839

*p < 0.05, **p < 0.01, ***p < 0.001. Note: Adjusted model includes controls for institution, year and field of study (and their interaction) parents’ country of origin, own country of origin, gender, age, age squared, parents’ educational level, number of roommates, home ownership, total proportion in quarantine at institution, alcohol consumption, use of public transport, total amount of offered teaching, and number of hours of paid work.

The unadjusted bivariate analysis yielded a negative association corresponding to a 41% reduction in the probability of COVID-19 (95% CI -78% to −5%) for two weeks of full time in-person teaching compared to full time online teaching. In the adjusted analysis, the association between in-person teaching and positive COVID-19 test remained negative, but was smaller and statistically non-significant (22% reduction, 95% CI -77%–33%, see Table 3).

The findings were similar for the other COVID-19 related outcomes, but the associations were weaker: In the unadjusted analyses, the associations for COVID-19 testing and quarantine were −26% (95% CI -32 to −19%), and −28% (95% CI -37% to −20%) respectively, for two weeks of full time in-person teaching compared to full time online teaching (see Table 3). In the adjusted analyses the corresponding associations were reduced to −4% (95% CI -13%–6%) and to −11% (95% CI -23%–1%).

For well-being and teaching satisfaction we found positive associations with in-person teaching in the unadjusted analyses, and the results remained statistically significant in the adjusted analyses: Without controlling, two weeks of in-person teaching was associated with a 12% (95% CI 11%–13%) higher well-being and a 24% (95% CI 23%–26%) higher teaching satisfaction, relative to two weeks of online teaching. In the adjusted analyses, the positive associations with in-person teaching were 7% for well-being (95% CI 6%–8%) and 16% for satisfaction with teaching (95% CI 14%–17%).

When we used actual campus presence as the independent variable instead of offered teaching modality, we found associations of comparable magnitude for well-being and teaching satisfaction (eFig. 1, Panel B). COVID-19 positive test, COVID-19 testing, and quarantine were all negatively associated with campus presence. This was as expected, since those with COVID-19 symptoms or in quarantine had stricter social distancing rules to adhere to.

### 3.2 Sensitivity analyses

The comparison between the quartiles with most and least in-person teaching (eFig. 2, Panel A), and the comparison of students with 80% or more in-person teaching against all other students (eFig. 2, Panel B), yielded results that were largely in line with our main findings.

The results for COVID-19 related outcomes were robust to lagging the outcome with one round (eFig. 3).

Neither replacing the proportion of students in quarantine with institution specific trends as a control variable, or controlling for county level COVID-19 incidences, or controlling for paid working time, led to substantially different results (data not shown).

The results from the lead model estimated by ordinary least squares (eFig. 1, Panel C) showed no significant effects for positive COVID-19 test, testing or quarantine, while there were significant lead effects for well-being and teaching satisfaction.

When we applied the fixed effects approach, the findings were largely compatible with the results from our main model (eTable 2 and eFig. 1, Panel D). All estimates for positive COVID-19 tests were substantially larger, but they remained statistically non-significant (p < 0.05). For quarantine and testing, the fixed effect approach yielded similar estimates as the main analyses. The estimates for well-being and teaching satisfaction were nearly halved in the fixed effects analyses, but they remained statistically significant. The difference between our main results and the fixed effect analyses, suggests that individuals more satisfied with life and with teaching tended to be in programs offering more in-person teaching.

We found no statistically significant lead effects for the fixed effects models (eFig. 1, Panel E).

Results from running our data through logit and negative binomial models were consistent with our main findings (data not shown).

## 4 Discussion

Our findings do not demonstrate a convincing association between teaching modality and COVID-19 risk among students in higher education. While the point estimate goes in the direction of a negative association between in-person teaching and COVID-19 risk, the lack of precision in our results means that we cannot rule out important effects in either direction.

However, we did find a relatively convincing positive association between in-person teaching and well-being, and between in-person teaching and teaching satisfaction. These findings are substantiated through a set of sensitivity analyses and do, in our judgement, provide evidence of downsides associated with shifting from in-person to online teaching. Still, the observational nature of our study means that we cannot ignore the risk that confounding may have biased the results.

Attempting to explain the apparent association between online teaching and COVID-19 risk, we hypothesised that online teaching leads to increased extracurricular activities that increase the risk of COVID-19. We therefore estimated the association between in-person teaching and social activities based on responses to the question, ”In the past 14 days, have you been to a social gathering where you would guess that there were 20 or more people?” The findings provided no support for our hypothesis: In-person teaching was associated with more, not less, social activities than was online teaching. This finding may suggest, however, that a shift to more in-person teaching is correlated with fewer social distancing interventions and behaviour in other domains, and that reductions in restrictions may have contributed to the effect on students’ improved well-being. Our attempts to adjust for this, e.g. by including the proportion of students in quarantine or the incidence of COVID-19 in the area in the model, may not have controlled sufficiently for confounding due to changes in restrictions. For teaching satisfaction, we believe it is unlikely that changes in social activity restrictions had an impact.

We employed two different analytical methods, i.e. ordinary least squares and fixed-effect multivariate regression. For the COVID-19 related outcomes, the difference between the results from the two methods is of limited interest, since they both yielded highly uncertain estimates with wide confidence intervals that bar us from drawing meaningful conclusions. The case is different for the two non-COVID-19 outcomes, well-being and teaching satisfaction. Here, both the ordinary least squares and fixed effects approaches yielded statistically significant positive associations with in-person teaching, but the estimates were substantially smaller in the fixed-effect model. We believe the fixed-effects model provides more credible estimates of the causal effect for these outcomes, since for the ordinary least squares model there were positive associations with in-person teaching also when outcomes came first (lead model). This was not the case for the fixed-effects model; however, this does not exclude the possibility that associations may be driven by correlations between exposures and outcomes at different time points (i.e. autocorrelation). The estimates from the fixed effects models were also the more conservative. Thus, a reasonable interpretation is that full time in-person teaching for two weeks was associated with a relative increase in well-being of 3% (95% CI 2%–4%), compared to full time online teaching, i.e. a difference of around 0.2 on the 0 to 10-scale used in the study. Correspondingly, our best estimate for teaching satisfaction was a 10% increase (95% CI 8%–11%).

We are not aware of other prospective observational studies using individual level data, than ours. Still, we were not able to generate conclusive results for the relationship between in-person teaching and COVID-19 risk. There are several reasons for this, most notably that the COVID-19 incidence was substantially lower than we expected, both in Norway generally, and among the participants specifically.

The critical lack of randomised trials in this area has been pointed out before [12,13]. For us, one key barrier for conducting such a study is the legal requirement in the Norwegian Health Research Act to obtain written informed consent from all who participate in health research. In practice, the demand for individual consent makes it impossible to carry out comparative studies where teaching institutions, municipalities, workplaces etc. are allocated to different forms of infection control measures [14].

## 5 Conclusion

We did not find clear evidence of an association between COVID-19 infection and teaching modality for students in higher education, but our findings indicate that shifting from in-person to online teaching may impact negatively on the students’ well-being.

## Declaration of interests

The authors declare that they have no known competing financial interests or personal relationships that could have appeared to influence the work reported in this paper.

The authors declare the following financial interests/personal relationships which may be considered as potential competing interests:

## Data Availability

After ensuring that re-identification is not possible, the dataset will be stored and made publicly available for at least 10 years at the Norwegian Centre for Research Data (NSD). We aim to have this in place by July 2021. Analytic codes are available upon request to the study chief analyst (RKH).
